# Molecular detection and phylogenetic analysis of *Mycoplasma gallisepticum* from backyard and commercial turkey flocks in Iran

**Published:** 2017-12-15

**Authors:** Saeed Rasoulinezhad, Mohammad Hassan Bozorgmehrifard, Hossein Hosseini, Nariman Sheikhi, Saeed Charkhkar

**Affiliations:** 1 *Department of Poultry and Obstetrics, Science and Research Branch, Islamic Azad University, Tehran, Iran; *; 2 *Department of Clinical Science, Faculty of Veterinary Medicine, Karaj Branch, Islamic Azad University, Alborz, Iran.*

**Keywords:** Iran, Molecular detection, *Mycoplasma gallisepticum*, Phylogenetic, Turkey

## Abstract

*Mycoplasma gallisepticum *(MG) is economically important pathogen of poultry causes airsacculitis and frequently infraorbital sinusitis in turkeys. Infections may remain without clinical signs, but they can make birds susceptible to secondary infections. This study was carried out for molecular detection and phylogenetic analysis of MG infections in commercial and backyard turkey flocks in some parts of Iran. A total number of 600 swab samples were collected from 18 commercial and 31 backyard turkey flocks. The PCR technique was performed for detecting 16S rRNA gene in the samples. Positive sample were subjected for sequencing of mgc2 gene. The results showed that 48.38% of backyard and 16.66% of commercial farms were positive for MG. These findings suggested the presence of MG in the commercial and backyard turkeys’ farms of Iran. The molecular analysis indicated high sequence similarity between some Iranian turkeys isolates with Indian and Pakistanian MG isolates. Furthermore, substitutions of MG nucleic acids and correlated amino acids sequences may lead to some antigenic modifications.

## Introduction

There are four species of *Mycoplasma* as pathogens in poultry. *Mycoplasma meleagridis* and *Mycoplasma iowae* are turkey specific pathogens and they cause airsacculitis, skeletal abnormalities and reduction in hatchability. *Mycoplasma synoviae* cause subclinical upper respiratory tract infection and infectious synovitis in turkey.^1^^,^^2^

*Mycoplasma gallisepticum* (MG) is an important pathogen of poultry that is responsible for elimination or downgrading of carcasses, increase in feed conversion ratio and economical loss of vaccination or drug prevention. MG is also commonly known as airsacculitis and often infra-orbital sinusitis causes in turkeys and chronic respiratory disease (CRD) in chickens with a wide variety of clinical signs.^3^^,^^4^ There are some reports of turkey neurological symptoms caused by MG.^5^

Infections caused by MG sometimes stay with no clinical signs, but make birds susceptible to secondary infections with bacteria such as *E.coli* and respiratory viruses like Newcastle disease virus in turkeys.^6^^,^^7^

Serological procedures are useful for flocks monitoring control programs but nonspecific reactors occur in some flocks which may cause false positive results.^8^^,^^9^ Moreover, the culture method for isolation of micro-organism is a time-consuming and non-accurate technique which may also be contaminated by environmental organisms.^10^

Gene-specific DNA probe is developed as an accurate molecular solution for the problems.^11^^-^^13^ Previous studies have shown that molecular methods are very useful to detect actual rate of MG in avian flocks, therefore, PCR technique is used during this study.^14^

Epidemiological investigations of MG in avian populations has focused mainly on the poultry farms. To our knowledge, there is not enough study regarding the prevalence and characterization of MG derived from backyard and industrial turkeys in Iran. Epidemiological studies according to phylogenetic of turkeys MG field isolates may indicate infection origin and relationship between different MG strains in other avian species, as the first step of control programs. Therefore, the aim of this study was to clarify the presence and genetic diversity of MG in turkeys in some provinces of Iran during the period from February to August 2016. 

## Materials and Methods

**Sample collection. **A total number of 600 tracheal, choanal cleft or/and infra orbital sinus swab samples were taken from 49 farms (31 backyard turkey farms and 18 commercial turkey farms). Swab samples were collected randomly from Tehran, Semnan, Isfahan, Qazvin, Zanjan, East Azerbaijan, Gilan, Mazandaran, and Golestan provinces in Iran during the period from February to August 2016 (All birds from each backyard farm and 20 birds from each house of commercial farms). Samples were transported immediately to the laboratory. 

**DNA extraction. **DNA was extracted from swab samples suspended in 1 ml of PCR-grade PBS. The suspensions were centrifuged for 30 min at 14,000 *g* at 4 ˚C. The supernatant was carefully removed and the pellets were suspended in 25 μL PCR-grade water. The tube and the contents were boiled for 10 min and then placed on ice for 10 min before centrifugation at 14,000 *g* for 5 min. The supernatant was used as DNA temples for PCR reactions. 

**Detection of MG 16s ribosomal RNA gene by PCR. **The PCR assay for detection of MG 16s ribosomal RNA gene carried out on all swab samples. The PCR reaction was performed in 50 μL reaction volume consisting of 5 μL 10X PCR buffer, 1.00 μL 10 mM dNTP, 0.50 μL of each primer (20 μM) MG-14F (5’-GAG CTA ATC TGTAAA GTT GGT C-3’) and MG-13R (5’-GCT TCC TTG CGG TTA GCA AC-3’), 0.25 μL Taq DNA polymerase, 2 μL 50 mM MgCl2 (Sinaclon, Tehran, Iran), 39.75 μL of deionized distilled water and 1 μL of template DNA.^15 ^The thermal cycle included three steps as follow: Primary denaturation was performed at 94 ˚C for 3 min as the first step. In the second step, 40 cycles each included three sections as denaturation at 94 ˚C for 30 sec, annealing at 55 ˚C for 30 sec and extension at 72 ˚C for 60 sec were performed. Eventually, final extension was conducted at 72 ˚C for 5 min as the third step.^15^ The PCR products were electrophoresed on 1.50% agarose gel for 1 hr at 100 V and visualized by staining with ethidium bromide. The S6 strain (Liverpool, UK) and ts-11 (Bioproperties, Ringwood, Australia) were used as positive control and distilled water as negative control in all PCR reactions.

**Detection of mgc2** **gene by PCR.** The second specific MG primers (Sinaclon), mgc2-2F (5'- CGC AAT TTG GTC CTA ATC CCC AAC A-3') and mgc2-2R (5'-TAA ACC CAC CTC CAG CTT TAT TTC C-3') were used for amplifying mgc2 gene (300bp).^16^^,^^17^ The mgc2-PCR was performed in a mixture with total volume of 25 μL, containing 2.5 μL of 10X PCR buffer, 2 μL of MgCl_2_ (50 mM), 0.20 μL of 10 mM dNTPs, 0.10 μL of each primer (50 pmol µL^-1^), 0.10 μL of *Taq* DNA polymerase (Sinaclon), 19 µL of deionized distilled water and 1 μL of extracted DNA as template. After denaturation at 95 ˚C for 1 min the reaction was performed in 40 cycles including denaturation (95 ˚C for 20 sec), annealing (60 ˚C for 40 sec), primary extension (72 ˚C for 10 sec) and a final extension at 72 ˚C for 5 min. All amplification reactions were performed in a thermal cycler (T100™; Bio-Rad Laboratories, Redmond, USA). Ethidium bromide-stained gel electrophoresis (1% agarose gel in 1X Tris-acetic acid-EDTA buffer) was visualized by UV transillumination system (Vilber Lourmat, Paris, France).

**Mgc2 gene sequencing and phylogenetic analysis. **Samples obtained from turkey flocks were first confirmed by PCR assays for detection of 16s rRNA. Then 11 purified PCR products of mgc2 gene were sequenced. The bidirectional sequencing (with the forward and reverse PCR primers) of PCR products was performed by Bioneer Inc. (Daejeon, South Korea). Homologies between nucleotide and amino acid sequences searched using BLAST and PSI-BLAST, respectively.^18^ Nucleotide and predicted amino acid sequences were aligned with ClustalW alignment and DNASIS protean software.^19^^,^^20^ Finally, the phylogenetic tree was constructed using MEGA MEGA software (version 6; Biodesign. Institute, Tempe, USA).^21^

## Results

Samples from 18 farms (three from commercial farms and 15 from backyard turkey farms) were positive for MG. In the other words, the 16s rRNA gene of MG was successfully amplified by PCR reactions in 36.73% of turkey farms (the amplicon size was 185 bp). The percentage of positive commercial and backyard turkey farms were 16.66% and 48.38%, respectively (Table 1). MG positive samples obtained from commercial farms were significantly lower than those found in backyard farms (*p* < 0.05, test of proportions).

**Fig. 1:**
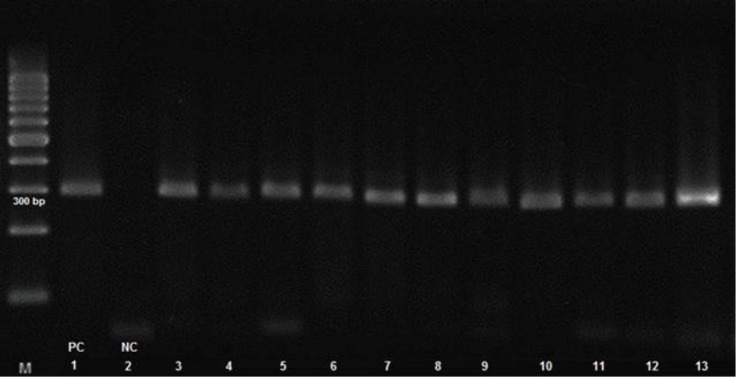
Mgc2 PCR result of 11 positive MG samples performed by electrophoresis. Lane M: 100 bp marker, Lanes 1: Positive control (PC) ts-11, Lanes 2: Negative control (NC), Lanes 3-13: Positive samples with 300 bp bands.

**Fig. 2:**
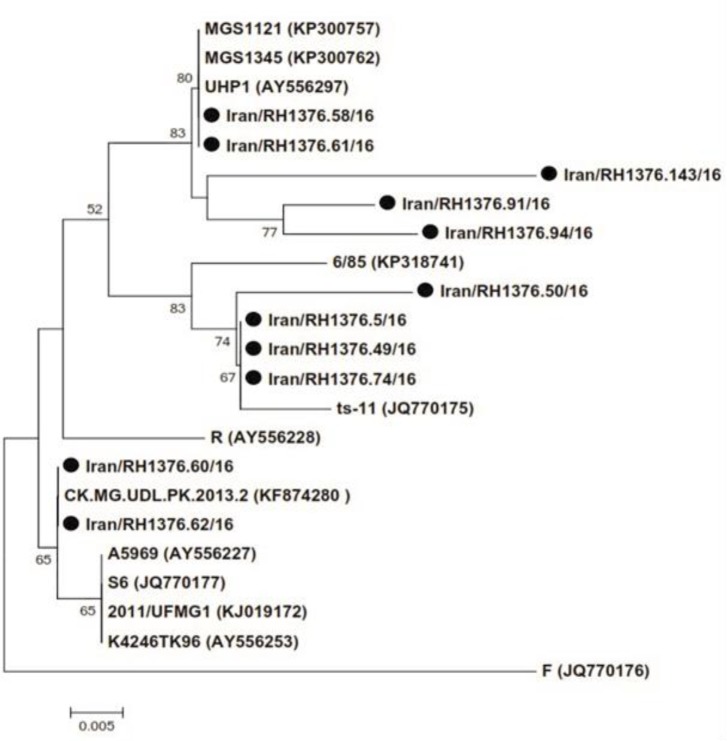
Phylogenetic tree of mgc2 gene based on the nucleotide sequences. A total number of 11 mgc2 genes were detected from turkey flocks of nine provinces of Iran and 12 mgc2 sequences obtained from GenBank.

The PCR results for mgc2 gene are shown in Figure 1. Based on the nucleotide phylogenetic tree of mgc2 gene, 11 positive samples were arranged in three main groups. We found that two Iranian turkey isolates; (RH1376.58/16 and RH1376.61/16) were quite similar to Indian strains and two strains (RH1376.60/16 and RH1376.62/16) were grouped with Pakistani strains showing more similarity. Also, we detected three isolates (RH1376.5/16, RH1376. 49/16 and RH1376.74/16) arranged in one group with 99.00% homogeneity in comparison to ts-11 vaccinal strain (Fig. 2). 

**Table 1:** Presence of *Mycoplasma gallisepticum *in backyard and commercial turkey farms in several provinces of Iran.

**Province**	**Number of commercial turkey farms**	**Number of backyard turkey farms**	**Total number of turkey farms**	**Number of positive commercial turkey farms**	**Number of positive backyard turkey farms**	**Total number of positive turkey farms**
**Qazvin**	1	6	7	1	3	4
**Gilan**	2	3	5	-	1	1
**Isfahan**	5	1	6	2	1	3
**Golestan**	1	8	9	-	4	4
**Mazandaran**	1	6	7	-	4	4
**Semnan**	1	2	3	-	1	1
**East Azerbaijan**	2	-	2	-	-	-
**Zanjan**	3	4	7	-	1	1
**Tehran**	2	1	3	-	-	-
**Total**	18	31	49	3	15	18

**Fig. 3:**
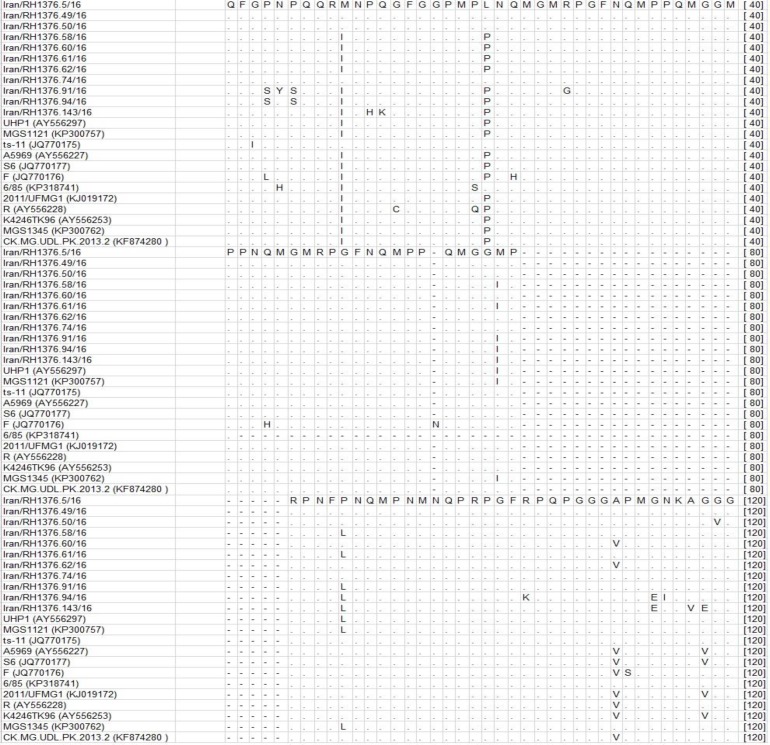
Amino acid sequence alignment of mgc2 gene. The alignment was done using ClustalW software. The sequences with Iran/RH in their names were isolated at present study. The other sequences were retrieved from GenBank database.

We identified substitutions in amino acid sequence of our isolates in comparison with each other. The position 10 was changed from methionine to isoleucine and in position 21 the leucine substituted by proline in seven isolates. Moreover, we found change of polar Methionine to nonpolar isoleucine and proline to leucine in five isolates in positions 62 and 90, respectively (Fig. 3). The partial mgc2 gene sequences were submitted to the GenBank data base under accession numbers KY651217– KY651227.

## Discussion

*Mycoplasma gallisepticum* is an important infectious disease that impresses commercial poultry production globally.^22^ It has been detected in many studies from poultry farms, but there is not enough data about presence of MG in turkey farms in Iran. Rezaie reported 2 (4.80%) commercial turkey flocks were positive via agglutination test with specific MG antiserum in East Azerbaijan province.^23^

Our findings demonstrated that the prevalence of MG in backyard turkey farms (48.38%) was significantly higher than commercial turkey farms (16.66%). It seems eradication programs in European breeder flocks as a main supplier of commercial poults ^24^ and mandatory control programs of Iranian veterinary organization for import of free pathogen poults are two reasons of lower prevalence of Iranian industrial farms. Michiels *et al*.^24^ showed all of the swab samples collected from turkey meat type in Belgium were negative by PCR. As well as in the Netherlands, there has been a decrease in positive flocks because of official surveillance program.^25^ It seems another reason of lower prevalence (36.73%) of MG in industrial farms in this condition is the role of principles of biosecurity during the breeding period. Although we know after placing poults in a polluted environment, they are infected and remain carriers.^22^

There were nine positive backyard flocks in the north of Iran including (Mazandaran, Golestan and Gilan provinces), which might be due to extensive backyard turkey rearing condition. It is well known that aerosol, temperature, atmospheric ammonia, dust and flock density all have important role in producing respiratory disease like mycoplasma.^7^^,^^26^ Some potential reservoirs of MG (carrier birds) are backyard flocks and there is increasing evidence that small backyard poultry flocks may be subclinicaly infected and transfer infection to commercial flocks.^27^^-^^29^ A high prevalence of MG infection in broiler has been reported by Seifi and Shirzad in this area.^30^

There were only two positive commercial farms (11.11%) in central parts of Iran (Isfahan province) which is probably due to proximity of multiage layer MG positive farms to turkey flocks. Prevalence of MG in layer flocks in central part of Iran has been reported. Haghighi-khoshkhoo *et al*. showed that 4 of 40 (10.00%) flocks were MG positive in this area.^31^ Also, the presence of MG in turkeys in central part of Iran may be due to the dry climate of Isfahan province. Dry weather increases transmission distance of aerosols, leading to increased susceptibility to infections.^32^ In the same way, the MG seroprevalence was slightly higher in the foothills (9.40%) than in coastal area (7.20%) in which humidity was greater.^30^

The results showed that two positive commercial farms (belonged to Isfahan and Tehran provinces), were suffering from respiratory distress, decreased feed intake, weight loss and increased mortality at the same time. Roussan *et al*.^33^ reported that all MG positive flocks were suffering from other respiratory disease(s) such as APV, MPV or NDV at the same time. According to higher number of positive backyard farms there were only one farm with respiratory distress and weight loss. 

Eleven positive samples for MG were grouped in five main cluster based phylogenetic of mgc2 gene. The high ratio of sequence similarity was observed between Iranian isolates with Pakestanian and Indian isolates, indicating the geographical distribution of MG strains between neighbor countries due to weak biosecurity strategies. 

The substitutions on positions 10 and 21 in seven of our turkeys isolates bring the new amino acids to the protein sequence making them more similar to a known pathogen strain S6 but more divergent to vaccine strain ts-11. We also recognized particular substitutions on few positions an exchange from polar methionine to nonpolar isoleucine and a change from proline to leucine in five isolates on positions 62 and 90, respectively. These variations demonstrated specific changes in this region of the mgc2 gene. The mgc2 gene is a well-protected gene and stimulant mucosal attachment process with encodes a cytadhesin protein.^34^^,^^35^ Antigenic membrane proteins were created by mgc2 gene can evolved fast and consequently allow MG to evade host immune system.^36^^,^^14^ Therefore, the low nucleotide difference between the targeted regions in this study may demonstrate mutagenesis in MG, a survival response from the pathogen for adaptation to new conditions in hosts body and immune system reactions. 

In conclusion, these findings suggested the molecular presence of MG in the commercial and backyard turkey farms, which might be due to extensive backyard turkey rearing condition without considering principles of biosecurity and/or supplying poults from local infected source. Based on the phylogenetic results and their distribution, some of the identified strains had close genetic relationship with Indian and Pakestanian MG strains, implicating the epidemio-geographical relation-ship between these isolates. 
